# Ten-Year Heterogeneity of Minimal Important Change and Patient Acceptable Symptom State After Lumbar Fusions

**DOI:** 10.1097/BRS.0000000000005065

**Published:** 2024-06-13

**Authors:** Leevi A. Toivonen, Jenna L.C. Laurén, Hannu Kautiainen, Arja H. Häkkinen, Marko H. Neva

**Affiliations:** a Department of Orthopedics and Traumatology, Tampere University Hospital, Tampere, Finland; b Faculty of Medicine and Health Technology, Tampere University, Tampere, Finland; c Primary Health Care Unit, Kuopio University Hospital, Kuopio, Finland; Folkhälsan Research Center, Helsinki, Finland; d Faculty of Sport and Health Sciences, University of Jyväskylä, Jyväskylä, Finland

**Keywords:** lumbar spine fusion, patient-reported outcomes, PROM, Minimal important change, MIC, patient acceptable symptom state, PASS

## Abstract

**Study Design.:**

Cohort study.

**Objective.:**

To evaluate heterogeneity (fluctuation) in minimal important change (MIC) and patient-acceptable symptom state (PASS) for patient-reported outcomes (PROMs) through 10 years after lumbar fusion.

**Summary of Background Data.:**

PROMs have become key determinants in spine surgery outcomes studies. MIC and PASS were established to aid PROM interpretations. However, their long-term stability has not yet been reported.

**Methods.:**

A consecutive series of elective lumbar fusions were followed up using the Oswestry Disability Index (ODI) and Visual Analogue Scale (VAS) for pain. Improvement was rated by a 4-point Likert scale into “improved” or “nonimproved.” Satisfaction-to-treatment was rated by the patients’ willingness to undergo surgery again. Receiver operating characteristics (ROC) curve analysis estimated MIC (95% confidence interval, CI) as the PROM change that best predicted improvement at distinct time-points. PASS (CI) was estimated as the lowest PROM score at which the patients were still satisfied. Heterogeneity across thresholds was evaluated using the DeLong algorithm.

**Results.:**

MIC for ODI represented heterogeneity across 10 years, ranging from −21 (−24 to −16) at two years to −8 (−7 to −4) at five years, P<0.001. The areas under the ROC curves (AUCs) (0.79 to 0.85) indicated acceptable to excellent discrimination. Heterogeneity was not significant in the MICs for the pain scores. At one year, MIC for back pain was −24 (−38 to −15), AUC 0.77, and for leg pain, it was −26 (−44 to −8), AUC 0.78. No significant heterogeneity was observed in 10-year PASS scores. At 1-year, PASS for ODI was 22 (15 to 29), AUC 0.85. Similarly, the one-year PASS for back pain was 38 (20 to 56), AUC 0.81, and for leg pain, it was 49 (26 to 72), AUC 0.81.

**Conclusions.:**

MIC for ODI fluctuated over 10 years after lumbar fusions. PASS values for all PROMs seemed most stable over time. Caution is needed when generic MIC values are used in long-term studies.

**Level of Evidence.:**

Level III

The patients’ experienced health status has become a key determinant in spine surgery outcomes studies. These largely rely on patient-reported outcome measures (PROMs) such as the Visual Analogue Scale (VAS) for pain, the Oswestry Disability Index (ODI) for disability, and various quality-of-life instruments.^1,2^

The interpretation of PROMs is however not unambiguous.^2,3^ Much effort has been invested in finding thresholds for a PROM change (ΔPROM) that best discriminates a clinically meaningful change in the health status. Various concepts and definitions surround these thresholds. Minimal clinically important difference (MCID) has been used to differentiate patients experiencing true improvement from those that remain unaffected.^4–7^ Minimal important change (MIC) is the threshold for ΔPROM that best predicts a patient perceiving improvement.^7^ However, these terms have been used inconsistently in the literature. MICs are frequently determined with anchor-based methods where ΔPROM is compared to a second, easily interpretable patient-graded measure.^2,5–8^ Patient-acceptable symptom state (PASS) is a newer cross-sectional concept. It represents the lowest PROM level at which patients are still satisfied with the treatment they received.^7,8^

In spine surgery settings, ΔPROM thresholds for minimal and substantial improvement have been defined for various populations and procedures.^9–13^ Schwartz *et al* reported instability in MCIDs for several PROMs over a one-year follow-up of disk herniation and spinal stenosis surgeries.^14^ Some reports have suggested PASS to be potentially less time-dependent than MIC.^8,15^ Longer follow-up periods are likely to be prone to greater bias.^2,16^ Yet, to the authors’ best knowledge, no studies have investigated the long-term trajectories of MIC or PASS for spine surgery outcomes.

The aims of the present study were to define MIC and PASS scores for ODI and pain scores and evaluate their possible fluctuation over a 10-year follow-up of lumbar fusions.

## MATERIALS AND METHODS

### Subjects and Surgeries

This study was based on prospectively collected data from 2 Finnish spine centers. All consenting patients undergoing elective lumbar fusion surgeries between 2008 and 2012 were enrolled in a follow-up study. In Finland, all fusion surgeries at the time were performed in public hospitals, making the study sample population-based. Only the patients who received their first fusion procedure were included in this study. Patients undergoing surgery for acute fractures, tumors, infections, or neuromuscular scoliosis were excluded. Those who had reoperations after the index surgery were not excluded.

Patients completed the demographical and clinical data. Surgeons recorded the surgical details. All surgeries were open, posterior fusions with pedicle screws, and necessary decompression. Interbody spacers were used at the surgeons’ discretion. All participants signed an informed consent. The ethical review boards of both study centers approved this study.

### Outcome Measures

Patients’ baseline status was collected before surgery, and follow-up data at 1, 2, 5, and 10 years were collected by mail. Study nurses reminded those who failed to complete the questionnaires. Also, participants with incomplete follow-up data were included. The Finnish validated version 2.0 of the Oswestry Disability Index (ODI) was used to measure back-related disability.^17,18^ ODI scores range between 0 and 100, with higher scores indicating greater disability and ODI <20 indicating minimal disability.^17^ The Visual Analogue Scale (VAS, 100 mm) was used to quantify back and leg pain.

Patients rated improvement by global assessment (anchor question) where they classified the results as (1) worse, (2), same, (3) better, or (4) much better as compared to baseline.^19^ Based on this, the patients were categorized as “not improved” or “improved.”

Satisfaction-to-treatment was graded by asking patients if they would go through the operation again now that they knew the course and the results.^20^ Satisfaction was dichotomized by “Yes” or “no”.

### Statistics

Summary statistics were described using means with SD, medians with interquartile range (IQR), or numbers as percentages. Longitudinal measures of PROMs were analyzed using a generalized estimating equations (GEE) model with an unstructured correlation structure. Receiver operating characteristic (ROC) curve analyses determined MICs (95% confidence intervals [CI]) as PROM change scores that best-predicted improvement at distinct time points. In addition, ROC curve analyses were used to estimate PASS (CI) as the lowest PROM score at which patients were still satisfied with the treatment received. The area under the curve (AUC) summarized the discriminative power of the models. A value of 0.70 to 0.80 was considered acceptable, and a value of >80 excellent.^21^ The best cutoff values were defined as the values with highest accuracy to maximize the Youden’s index (sensitivity + specificity −1). For the corresponding diagnostic characteristics, sensitivity, specificity, and positive likelihood ratio, 95% CIs were obtained using bias-corrected bootstrapping (5000 replications). Heterogeneity between the thresholds was evaluated using the DeLong algorithm. Sample size calculations were based on AUC values.^22^ A sample size of ~200 patients was required to detect differences in AUCs of 0.70 to 0.80, delta 0.10 (=0.05, power=90%). Stata 18 (StataCorp LP, College Station, TX, USA) statistical package was used for the analyses.

## RESULTS

A total of 613 patients met the inclusion criteria. Most were female (69%), were operated for spinal stenosis with degenerative spondylolisthesis (53%), and received short fusion (69%) (Table 1). During the 10-year study period, 80 (13%) patients were deceased. Of those alive, 95% completed PROMs at one year, and still 73% at 10 years.

**TABLE 1:** Baseline Patient Demographics and Clinical Data (n=613)

	Measurement
Women, n (%)	421 (69)
Age, years, mean (SD)	61 (12)
BMI, mean (SD)	27.9 (4.4)
Education years*, mean (SD)	11.7 (3.8)
Cohabiting*, n (%)	401 (66)
Retired*, n (%)	349 (57)
Duration of spinal complaint*, years, median (IQR)	10 (4, 20)
Comorbidities*, n (%)	
Cardiovascular	323 (53)
Diabetes	72 (12)
Rheumatic	61 (10)
Psychiatric	22 (4)
Pulmonary	18 (3)
Neurologic	17 (3)
Cancer	10 (2)
Alcohol consumption per week*, doses, median (IQR)	1 (0, 3)
Smoking*, n (%)	93 (15)
Diagnosis, n (%)	
Spinal stenosis with degenerative spondylolisthesis	327 (53)
Spinal stenosis without degenerative spondylolisthesis	96 (16)
Isthmic spondylolisthesis	93 (15)
Degenerative disc disease	51 (8)
Deformity	46 (8)
Fusion length, n (%)	
1 to 2 levels	425 (69)
>2 levels	187 (31)
Interbody device, n (%)	123 (20)

* Self-reported data.

BMI indicates body mass index; IQR, interquartile range; SD, standard deviation.

### MIC Values

**Figure 1:**
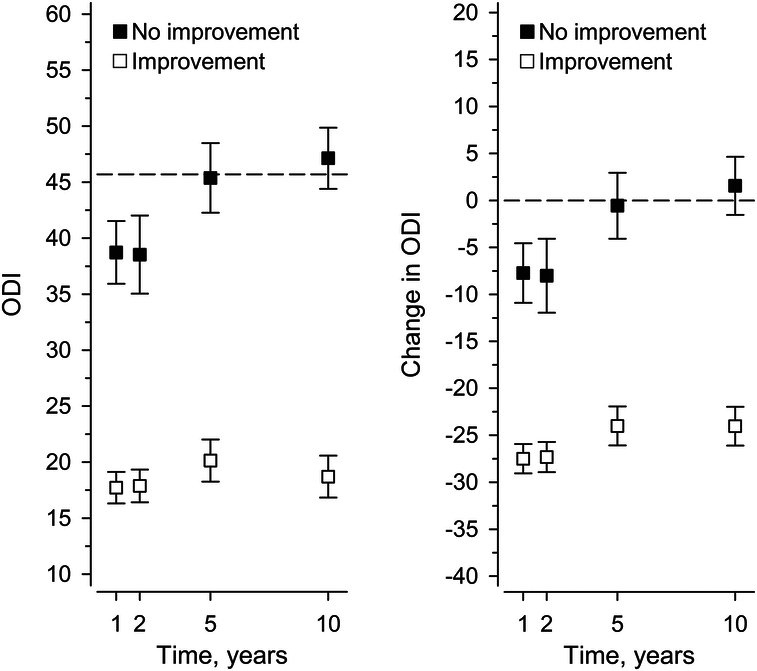
Mean and change scores (whiskers, 95% confidence intervals) of the Oswestry disability index (ODI) by self-rated improvement status. The dashed line represents the baseline status.

**Figure 2:**
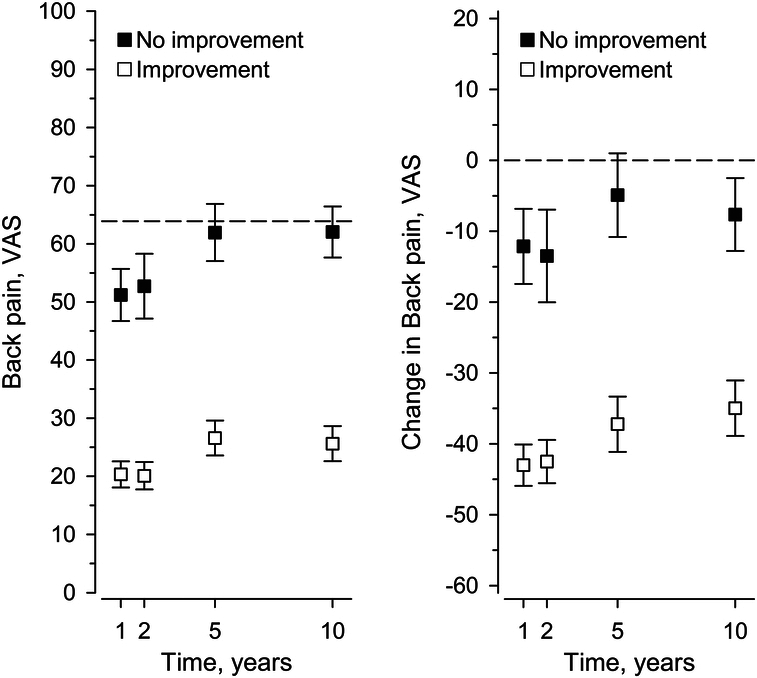
Mean and change scores (whiskers, 95% confidence intervals) of the Visual analog scale (VAS) for back pain by self-rated improvement status. The dashed line represents the baseline status.

**Figure 3:**
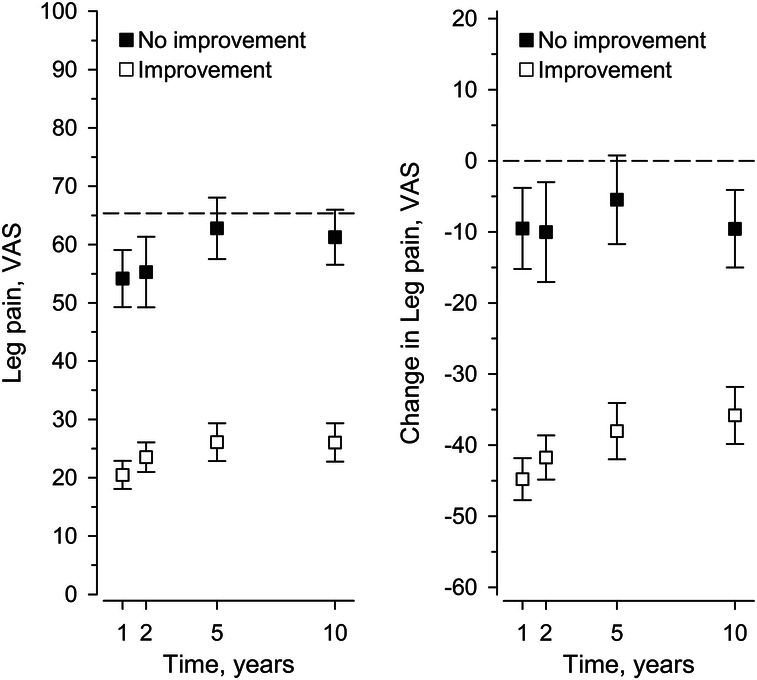
Mean and change scores (whiskers, 95% confidence intervals) of the Visual analog scale (VAS) for leg pain by self-rated improvement status. The dashed line represents the baseline status.

At one year, 80% of the patients perceived themselves improved. This rate increased to 85% at two years but was reversed to a slight decrease after that, remaining still at 68% at 10 years. The ODI and VAS trajectories markedly diverged between the improved and nonimproved cohorts (Figs. 1–3). The 1-year MIC for ODI of −20 (95% confidence interval, CI: −24 to −16) over halved by 5 years [−8 (95 CI: (−17 to −4)], representing significant heterogeneity, *P*=0.003 (Table 2). That is, MIC for ODI fluctuated over time, mainly directed downward. The areas under the ROC curves (AUC) (0.79 to 0.85) indicated acceptable to excellent discrimination. In contrast to ODI, heterogeneity remained insignificant in MIC trajectories for back or leg pain (*P*=0.28, *P*=0.64, respectively), even though there was a trend toward instability. AUC (0.73 to 0.78) indicated acceptable discrimination.

**TABLE 2:** Minimal Important Change (MIC) Values for the Oswestry Disability Index (ODI) and Visual Analogue Scale (VAS) for Back and Leg Pain

		Diagnostic characteristics					
	MIC	AUC	Sensitivity	Specificity	LR positive	OR	Accuracy, %
Time	(95% CI)	(95% CI)	(95% CI)	(95% CI)	(95% CI)	(95% CI)	(95% CI)
ODI							
1 yr	−20 (−24 to −16)	0.79 (0.75 to 0.84)	0.70 (0.65 to 0.74)	0.77 (0.68 to 0.84)	3.05 (2.18 to 4.27)	7.78 (4.86 to 12.44)	71 (68 to 75)
2 yr	−21 (−29 to −13)	0.81 (0.76 to 0.87)	0.64 (0.59 to 0.69)	0.86 (0.76 to 0.93)	4.42 (2.55 to 7.67)	10.49 (5.43 to 20.25)	67 (63 to 71)
5 yr	−8 (−17 to −4)	0.85 (0.80 to 0.90)	0.85 (0.80 to 0.90)	0.69 (0.58 to 0.78)	2.76 (2.04 to 3.73)	13.75 (7.90 to 23.95)	82 (77 to 85)
10 yr	−10 (−17 to −3)	0.85 (0.80 to 0.89)	0.81 (0.76 to 0.86)	0.74 (0.66 to 0.82)	3.16 (2.33 to 4.28)	12.61 (7.60 to 20.93)	79 (75 to 83)
*P*-value*	0.003						
Back pain, VAS							
1 yr	−24 (−38 to −15)	0.77 (0.72 to 0.82)	0.72 (0.67 to 0.76)	0.73 (0.64 to 0.81)	2.70 (1.97 to 3.68)	6.97 (4.39 to 11.06)	72 (68 to 76)
2 yr	−34 (−45 to −21)	0.77 (0.71 to 0.82)	0.63 (0.58 to 0.67)	0.81 (0.70 to 0.89)	3.32 (2.06 to 5.35)	7.23 (3.94 to 13.27)	66 (61 to 70)
5 yr	−17 (−36 to −6)	0.77 (0.72 to 0.82)	0.72 (0.66 to 0.78)	0.69 (0.59 to 0.78)	2.35 (1.71 to 3.23)	5.88 (3.50 to 9.87)	72 (67 to 76)
10 yr	−22 (−28 to −15)	0.74 (0.68 to 0.79)	0.66 (0.60 to 0.72)	0.76 (0.67 to 0.83)	2.74 (1.97 to 3.80)	6.13 (3.76 to 10.00)	69 (65 to 74)
*P*-value*	0.28						
Leg pain, VAS							
1 yr	−26 (−44 to −8)	0.78 (0.73 to 0.83)	0.74 (0.70 to 0.78)	0.70 (0.60 to 0.78)	2.44 (1.83 to 3.26)	6.51 (4.12 to 10.27)	73 (69 to 77)
2 yr	−18 (−40 to −11)	0.77 (0.72 to 0.83)	0.76 (0.72 to 0.80)	0.69 (0.57 to 0.80)	2.49 (1.75 to 3.55)	7.28 (4.22 to 12.56)	75 (71 to 79)
5 yr	−20 (−29 to −6)	0.78 (0.72 to 0.83)	0.71 (0.65 to 0.76)	0.77 (0.67 to 0.85)	3.08 (2.10 to 4.51)	8.15 (4.68 to 14.18)	73 (68 to 77)
10 yr	−11 (−34 to −4)	0.73 (0.67 to 0.78)	0.77 (0.72 to 0.82)	0.58 (0.49 to 0.67)	1.84 (1.48 to 2.30)	4.73 (2.96 to 7.56)	71 (66 to 76)
*P*-value*	0.64						

MIC represents the best cutoff value in ODI change to discriminate between improvement and nonimprovement.

* *P*-value for heterogeneity (fluctuation over time) of MIC

AUC indicates area under the curve; CI, confidence interval; LR, likelihood ratio; OR, odds ratio.

**Figure 4:**
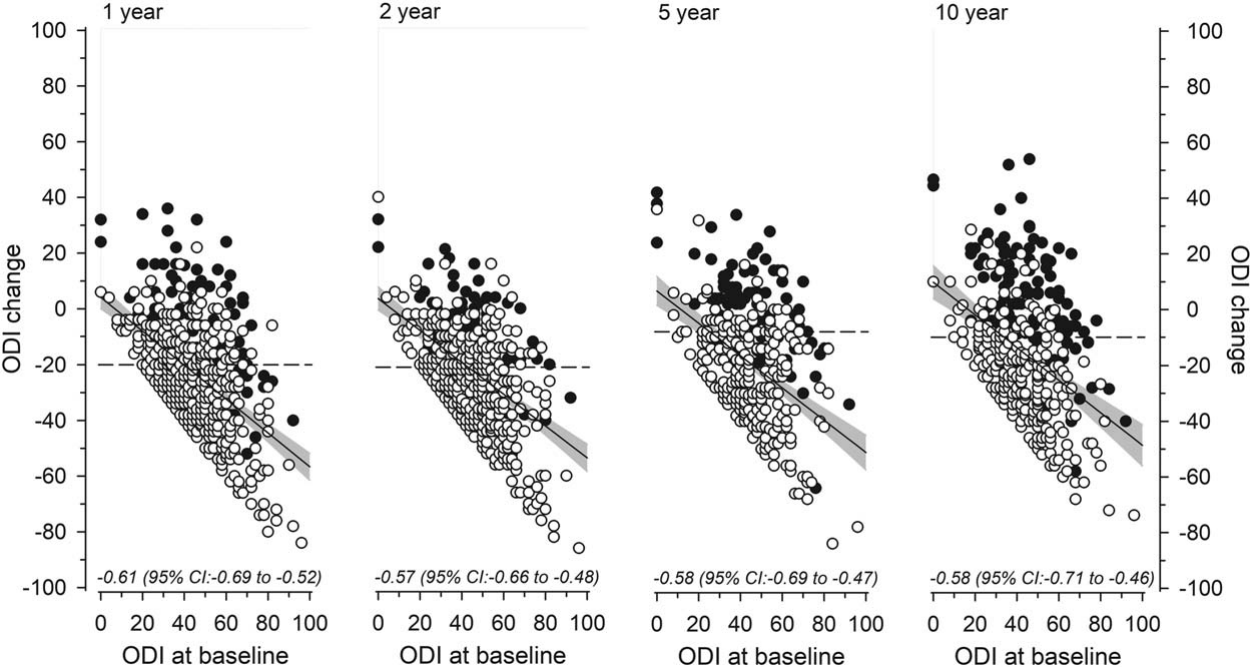
Scatterplots illustrating how baseline Oswestry Disability Index (ODI) scores are reflected on ODI change scores at follow-up. White circles describe self-reportedly improved and black circles nonimproved individuals. Oblique lines represent regression coefficients (shadow areas, their 95% confidence intervals) between baseline disability and self-reported improvement at follow-up. Dashed lines represent Minimal important change (MIC) values that best discriminate between improvement and nonimprovement at follow-up.

Figure 4 illustrates the relationship between baseline disability and self-reported improvement by ODI at follow-up. Greater baseline disability was associated with greater postoperative improvement.

### PASS Values

**Figure 5:**
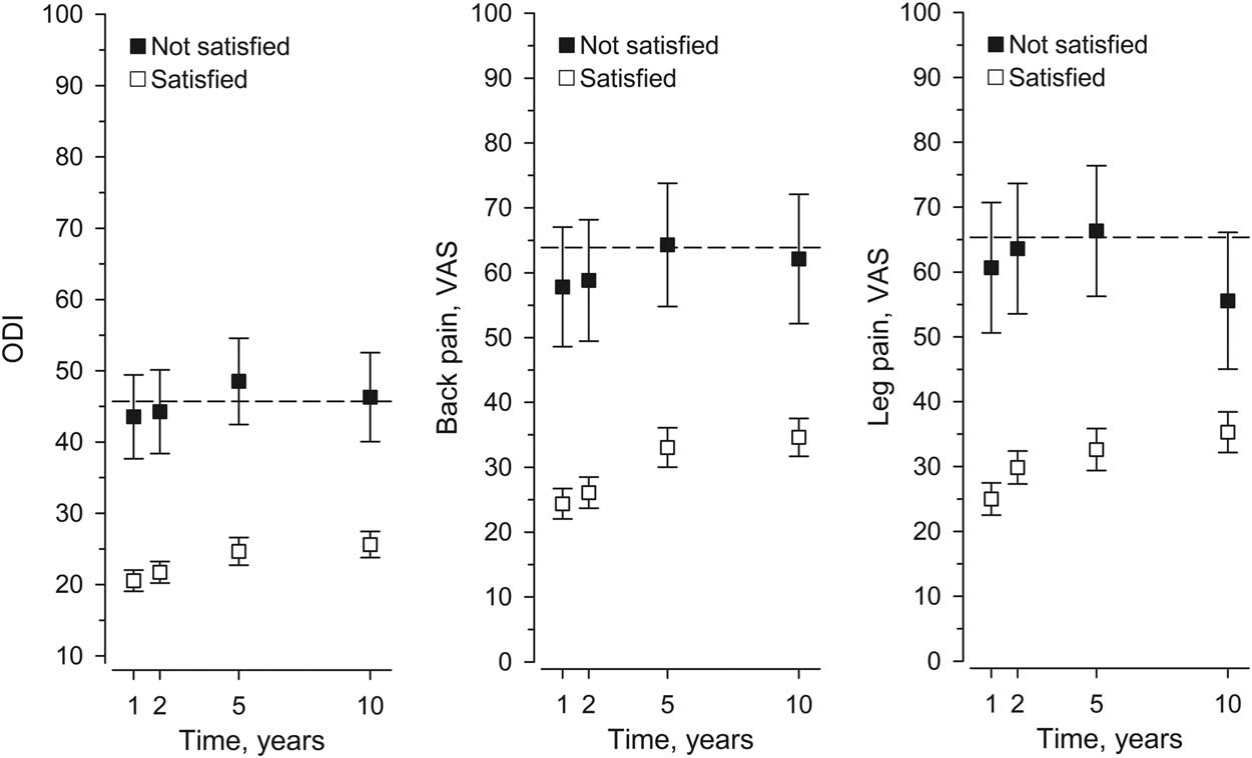
Mean Oswestry disability index (ODI) and Visual analog scale (VAS) scores by satisfaction-to-treatment status. The dashed lines represent the baseline status.

At one year, 94% of the patients were deemed satisfied-to-treatment owing to that they would have undergone surgery again. This rate remained over 90% throughout follow-up. ODI and VAS trajectories diverged by satisfaction status (Fig. 5). Heterogeneity across PASS scores remained insignificant for all PROMs (Table 3). PASS for ODI stayed at 22 except for the insignificant 5-year deviation to 38 (26 to 48), *P*=0.059. AUC ranged from 0.79 to 0.85, indicating acceptable to excellent discrimination. PASS for back pain and leg pain remained steadily around 40, *P*=0.91 and *P*=0.92, respectively. AUC ranged from 0.69 to 0.81, indicating acceptable to excellent discrimination except for poor discrimination in 10-year leg pain.

**TABLE 3:** Patient Acceptable Symptom State (PASS) Values for the Oswestry Disability Index (ODI) and Visual Analogue Scale (VAS) for Back and Leg Pain

		Diagnostic characteristics					
	PASS	AUC	Sensitivity	Specificity	LR positive	OR	Accuracy, %
Time	(95% CI)	(95% CI)	(95% CI)	(95% CI)	(95% CI)	(95% CI)	(95% CI)
ODI							
1 yr	22 (15 to 29)	0.85 (0.80 to 0.89)	0.63 (0.59 to 0.67)	0.97 (0.85 to 1.00)	21.96 (3.18 to 151.70)	57.23 (9.82 to >100)	65 (61 to 69)
2 yr	22 (8 to 36)	0.83 (0.77 to 0.89)	0.59 (0.55 to 0.64)	0.91 (0.77 to 0.98)	6.92 (2.34 to 20.47)	15.55 (4.99 to 48.35)	61 (57 to 65)
5 yr	38 (28 to 48)	0.81 (0.73 to 0.89)	0.76 (0.71 to 0.81)	0.79 (0.61 to 0.91)	3.60 (1.86 to 6.97)	12.02 (5.12 to 28.15)	77 (72 to 81)
10 yr	22 (9 to 35)	0.79 (0.72 to 0.86)	0.52 (0.47 to 0.58)	0.97 (0.83 to 1.00)	16.24 (2.36 to 111.97)	33.02 (5.63 to 60.41)	56 (51 to 61)
*P*-value*	0.059						
Back pain, VAS							
1 yr	38 (20 to 56)	0.81 (0.74 to 0.88)	0.75 (0.71 to 0.78)	0.80 (0.63 to 0.92)	3.73 (1.92 to 7.24)	11.71 (5.11 to 26.81)	75 (71 to 78)
2 yr	39 (24 to 54)	0.78 (0.69 to 0.87)	0.72 (0.68 to 0.76)	0.79 (0.62 to 0.91)	3.51 (1.81 to 6.80)	10.02 (4.36 to 22.99)	73 (69 to 76)
5 yr	47 (25 to 70)	0.73 (69 to 77)	0.73 (71 to 75)	0.73 (0.65 to 0.80)	1.80 (1.44 to 2.26)	4.77 (3.00 to 7.60)	73 (71 to 75)
10 yr	45 (18 to 72)	0.76 (0.68 to 0.84)	0.62 (0.57 to 0.67)	0.80 (0.61 to 0.92)	3.11 (1.51 to 6.39)	6.58 (2.69 to 16.05)	64 (59 to 68)
*P*-value*	0.91						
Leg pain, VAS							
1 yr	49 (26 to 72)	0.81 (0.74 to 0.88)	0.79 (0.75 to 0.82)	0.76 (0.58 to 0.89)	3.25 (1.78 to 5.96)	11.68 (5.22 to 26.07)	79 (75 to 82)
2 yr	46 (21 to 71)	0.79 (0.72 to 0.87)	0.69 (0.65 to 0.73)	0.79 (0.61 to 0.91)	3.25 (1.68 to 6.29)	8.24 (3.58 to 18.95)	70 (66 to 73)
5 yr	43 (8 to 78)	0.79 (0.71 to 0.86)	0.63 (0.57 to 0.68)	0.82 (0.65 to 0.93)	3.47 (1.67 to 7.18)	7.67 (3.15 to 18.60)	65 (60 to 70)
10 yr	37 (11 to 63)	0.69 (0.60 to 0.77)	0.56 (0.50 to 0.61)	0.80 (0.61 to 0.92)	2.79 (1.36 to 5.75)	5.07 (2.07 to 12.36)	58 (53 to 63)
*P*-value*	0.92						

PASS represents the optimal cutoff.

* *P*-value for heterogeneity (fluctuation over time) of PASS.

AUC indicates area under the curve; CI, confidence interval; LR, likelihood ratio; OR, odds ratio.

## DISCUSSION

The present study demonstrated heterogeneity (ie, fluctuation) in the 10-year trajectory of MIC for ODI after lumbar fusions. Heterogeneity remained insignificant with the VAS scores. Overall, PASS scores seemed more stable over time.

### MIC Values

The present one-year MIC for ODI of −20 was modestly higher than the most cited MCID value for ODI of −12.7.^9^ The study by Copay *et al* ^9^ discriminated between those with no change and minimal change. Our anchor method discriminated between improvement and nonimprovement. This may be a clinically more relevant demarcation line, but also produce higher threshold values. Prior literature has reported a wide spectrum of potential MCID values depending on calculation methods, sample characteristics, specific pathologies, and duration of follow-up.^8,10–12^

The present one-year MIC values for back and leg pain of −24 and −26 were higher than the MCID values translated from Copay *et al* ^9^ (−12 and −16). These were defined using distribution-based methods, but an anchor-based approach would have resulted in values of −25 and −15. The study of Glassman *et al* ^10^ defined substantial clinical improvement thresholds at −25. Those were consistent with the present results. There was a trend toward instability, but that remained insignificant with the VAS scores.

To date, no study has reported the long-term stability of MIC values. Using anchor-based methods, in which patients compare their changed health status over long periods, causes bias. Recall bias (patients incorrectly remember their prior health status),^2,16,23^ response shift (patients adapt to their changed health status),^2,16^ and external confounding from other pathologies as well as aging all skew long-term ΔPROM evaluations. Advancing age presumptively increases physical degradation and may also increase recall bias, which all are projected to PROMs. In the present study, the mean health status among both improved and nonimproved patients decreased over the course of follow-up, reflecting the recurrence of complaints in some individuals. Along that, the demarcation line between improved and nonimproved patients was lowered. We assume the patients’ fading recollection of their previous performance, along with aging, played a key role here.

### PASS Values

Our PASS for ODI of 22 (except at five years) was similar to the one- and two-year values reported by van Hooff *et al*.^15^ Goh *et al* ^24^ reported the 6-month and 2-year values of 18 and 15, respectively. The insignificant five-year deviation was likely a statistical artifact due to the low proportion of nonsatisfied respondents at five years.

The present PASS values for VAS around 40 were aligned with those reported by Daste *et al* on chronic low back pain patients (40 to 50) and Mannion *et al* ^25^ in a spinal deformity cohort (30). The latter was increased to 49 per higher baseline pain or age of ≥50 years. Greater baseline symptoms may yield satisfaction with higher residual symptoms, given patients’ preoperative adaptation to their functional limitations.^24,26^ Also, in our sample, greater baseline disability signaled greater improvements throughout follow-up.

Generally, PASS for ODI seemed lower than PASS for pain scores, given that ODI ≤20 indicates minimal disability.^17^ This concurs with prior reports that patients may tolerate functional deficits better than pain.^27^

Our discovery of greater stability in PASS than in MIC values was echoed by suggestions from prior literature.^8,15^ Interestingly, the satisfaction rates were higher than the improvement rates throughout follow-up. That discrepancy assumably stems from the anchor questions used, although at follow-up, patients may increasingly value benefits achieved in the early postoperative years despite the potential dimming of achievements.

### Limitations

The length of follow-up, prospectively collected data, and the adequately powered and population-based sample are strengths of the present study. The declining long-term response rates constitute a limitation of this study, as is typical for long-term follow-ups. However, some reports have estimated attrition bias from nonrespondents limited.^28–30^ Aging inherently influences long-term responses. The lack of a general population control cohort prevents us from determining the role of aging. We chose not to exclude patients with missing measures as our goal was to analyze general MIC and PASS values from a comprehensive real-life sample across distinct time points rather than change them within individual patients. Therefore, patients who had undergone further surgeries were also included. Thus, we can hear the voice of a more representative sample of lumbar fusion patients.

As stated above, the characteristics of the samples used influence MIC and PASS values. Moreover, there are no established criteria for the anchor questions. We measured satisfaction-to-treatment by willingness to undergo surgery again in a situation analogous to the baseline. This can be regarded as the ultimate measure of satisfaction. A variety of anchor questions may reduce the comparability across studies. However, these issues do not negate our finding of the long-term instability of MIC for ODI. The present findings shine a light on the difficulties in delineating satisfactory long-term outcomes. Many studies interpret PROM changes by using MIC values (or their equivalents) derived from other populations and calculated at single time points. These analyses need to be interpreted carefully, and extra caution is needed when evaluating long-term treatment effects.

## CONCLUSIONS

MIC for ODI showed significant heterogeneity (fluctuation) over ten years after lumbar fusions. Instability was not significant in MIC for pain scores. PASS values seemed most stable over time. Caution should be exercised when using generic MIC values in interpreting long-term outcomes.

Key Points

- MIC and PASS are concepts developed to delineate favorable outcomes of surgery.
- This cohort study used anchor-based methods to evaluate heterogeneity (fluctuation) in MIC and PASS over the 10-year follow-up after lumbar fusions.
- Heterogeneity was significant in MIC for ODI. PASS values seemed more stable than MIC.
